# Supplementary figures and images for: Evaluation of sequence-based tools to gather more insight into the positioning of rhizogenic agrobacteria within the Agrobacterium tumefaciens species complex
Source: PLoS One. 2024 Nov 19;19(11):e0302954. doi: 10.1371/journal.pone.0302954 (PMC11575935; doi:10.1371/journal.pone.0302954)

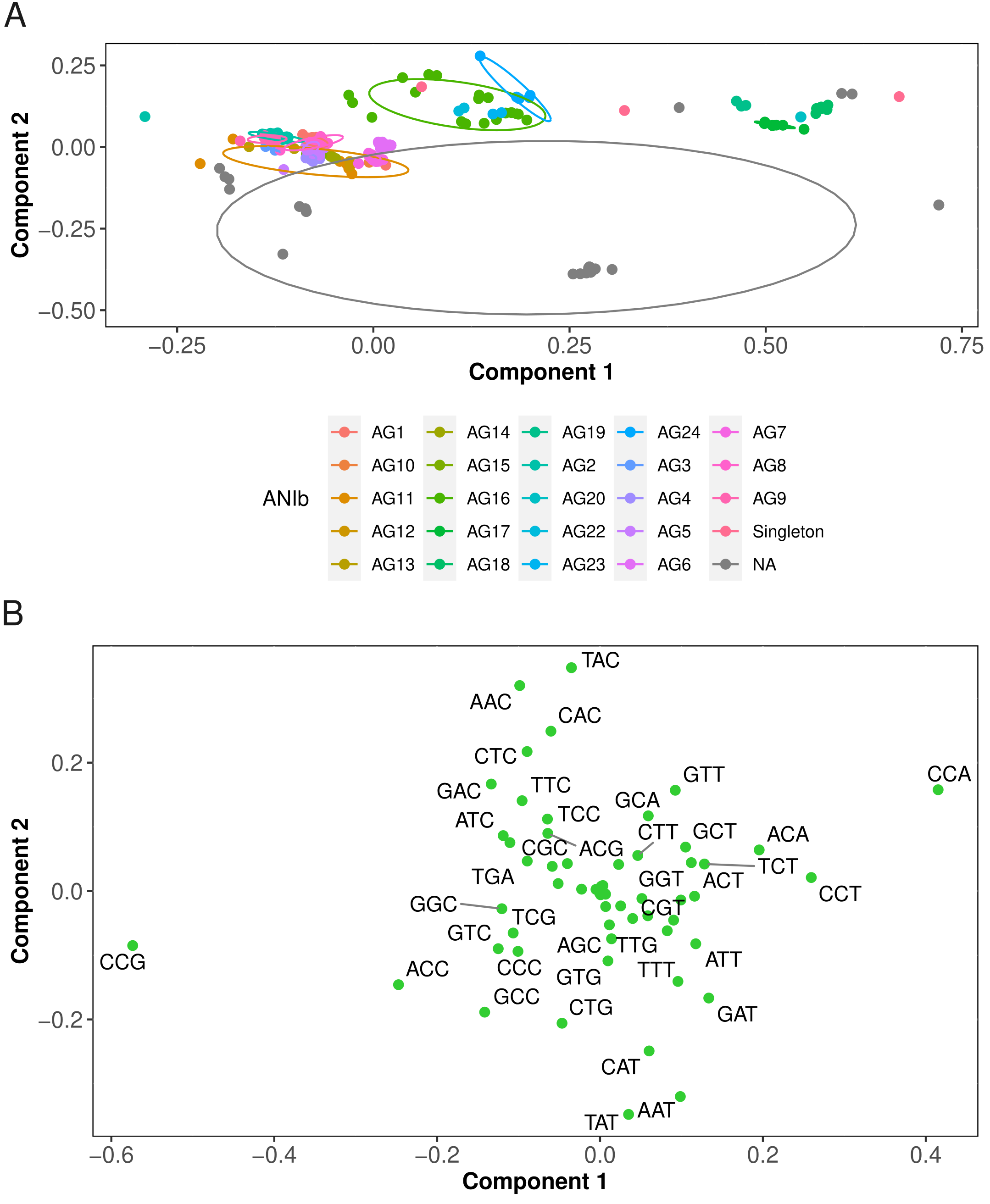

Supplement: S4 Fig — (A) Scores plots showing the distribution of the Atsc genomes analyzed in this study according to the first two components (which explained 79.7% of the total variance) obtained by principal component analysis (PCA) of the normalized relative synonymous codon usage (NRSCU) values. Colors of points and the 68% data concentration ellipses denote different ANIb groups. (B) PCA loading plot, where each dot represents the loadings on the first two principal components for one factor (NRSCU for a particular codon). (TIFF) [file pone.0302954.s004.tiff]
